# Physiological tissue-specific and age-related reduction of mouse TDP-43 levels is regulated by epigenetic modifications

**DOI:** 10.1242/dmm.049032

**Published:** 2022-04-29

**Authors:** Miriam Pacetti, Laura De Conti, Luciano E. Marasco, Maurizio Romano, Mohammad M. Rashid, Martina Nubiè, Francisco E. Baralle, Marco Baralle

**Affiliations:** 1 RNA Biology, International Centre for Genetic Engineering and Biotechnology (ICGEB), Padriciano 99, 34149 Trieste, Italy; 2 Universidad de Buenos Aires (UBA), Facultad de Ciencias Exactas y Naturales, Departamento de Fisiología, Biología Molecular y Celular and CONICET-UBA, Instituto de Fisiología, Biología Molecular y Neurociencias (IFIBYNE), CP1428 Buenos Aires, Argentina; 3 Department of Life Sciences, Via Valerio 28, University of Trieste, 34127 Trieste, Italy; 4 Fondazione Italiana Fegato-Onlus, Bldg. Q, AREA Science Park, ss14, Km 163.5, Basovizza, 34149 Trieste, Italy

**Keywords:** Amyotrophic lateral sclerosis, Epigenetics, TDP-43, *TARDBP*, RNA-binding proteins, Aggregation, RNA

## Abstract

The cellular level of TDP-43 (also known as TARDBP) is tightly regulated; increases or decreases in TDP-43 have deleterious effects in cells. The predominant mechanism responsible for the regulation of the level of TDP-43 is an autoregulatory negative feedback loop. In this study, we identified an *in vivo* cause-effect relationship between *Tardbp* gene promoter methylation and specific histone modification and the TDP-43 level in tissues of mice at two different ages. Furthermore, epigenetic control was observed in mouse and human cultured cell lines. In amyotrophic lateral sclerosis, the formation of TDP-43-containing brain inclusions removes functional protein from the system. This phenomenon is continuous but compensated by newly synthesized protein. The balance between sequestration and new synthesis might become critical with ageing, if accompanied by an epigenetic modification-regulated decrease in newly synthesized TDP-43. Sequestration by aggregates would then decrease the amount of functional TDP-43 to a level lower than those needed by the cell and thereby trigger the onset of symptoms.

## Abstract (precis)

**Summary:** Identification of a cause-effect relationship between epigenetic modifications that occur on the promoter and histones of mouse *TARDBP* and the level of TDP-43 both in tissues and in cell culture.

## INTRODUCTION

RNA-binding protein (RBP) family members play essential and diverse roles in RNA processing (Singh et al., 2015), and variations in their levels have a wide impact on the overall cellular metabolism. An alteration in the stoichiometry of the interaction between an RBP and its target can lead to severe dysfunction and pathological phenotypes. For example, impaired RBP expression, cellular mislocalization, aggregation or sequestration results in the onset of neurological disorders (Conlon and Manley, 2017; De Conti et al., 2017). Several variations in RBP cellular levels have been observed in both physiological and pathological settings. This variation can be due to the regulation of their expression levels, to their localization and to their splicing. One of the most impressive sets of variations is observed during development (Baralle and Giudice, 2017). This phenomenon provides potential therapeutic pathways for many diseases, particularly those in which the overexpression or underexpression of an RBP might prove beneficial for the pathology (Nussbacher et al., 2019). However, a detailed analysis of specific RBP behaviour in different tissues during the lifetime of an organism, and of the molecular mechanisms involved in modulating the variations in the levels of specific RBPs, is lacking.

TAR DNA-binding protein (TDP-43, also known as TARDBP) is a heterogeneous ribonucleoprotein (hnRNP) encoded by the *TARDBP* gene, and its sequence has been highly conserved throughout evolution. The cellular level of TDP-43 is tightly regulated. Indeed, as demonstrated experimentally, increasing or decreasing the level of TDP-43 causes defects in the general RNA homeostasis in cells (Ratti and Buratti, 2016). In particular, in neurons, the depletion of TDP-43 via aggregation is well known for resulting in neuronal alteration and/or loss, resulting in amyotrophic lateral sclerosis (ALS) (Baralle et al., 2013). We previously identified the predominant mechanism responsible for the regulation of the level of TDP-43. A high level of TDP-43 activates an autoregulatory negative feedback loop mediated via a region located in its 3′ untranslated region (Avendaño-Vázquez et al., 2012; Ayala et al., 2011; Bembich et al., 2014). However, a northern blot analysis showed that although ubiquitously expressed, levels of TDP-43 vary considerably among different human tissues (Buratti et al., 2001). Furthermore, we observed an age-related decrease in the *Drosophila* TDP-43 homologue TBPH in the brain (Cragnaz et al., 2015). Moreover, the same study provided data showing a similar phenomenon previously observed in mice (Huang et al., 2010; Liu et al., 2015), which suggests the existence of an evolutionarily conserved programme for reducing the level of TDP-43. Additionally, the level of TDP-43 in mice is regulated during embryonic development (Sephton et al., 2010).

These observations suggest the involvement of other mechanisms, in addition to autoregulation, in the control of TDP-43 expression. The physiological selective reduction of TDP-43 levels in tissues, such as the brain, might influence the time of onset of symptoms in pathologies, such as ALS and frontotemporal dementia. This effect might be further enhanced in the presence of concomitant additional factors, such as protein misfolding and insolubility. In this study, we investigated the physiological TDP-43 level variations in different mouse tissues at 10 and 90 days. We observed a tissue-specific pattern of expression; with increasing age, a marked reduction was found in some tissues, whereas other tissues showed constant levels.

Epigenetic modifications are well known for regulating transcription by acting directly on DNA and histone tails (Berger, 2007; Bernstein et al., 2007). In the mammalian genome, the most common epigenetic modification of DNA is the covalent addition of a methyl group to position 5 of cytosine in the context of the palindromic dinucleotide CpG (Illingworth and Bird, 2009). This modification is established and maintained by a family of DNMTs (Law and Jacobsen, 2010), and when it occurs in gene promoter regions it is associated with transcriptional repression (Greenberg and Bourc'his, 2019). The vast majority of DNA methylation data available are related to cancer or development; however, in physiological conditions, several studies, including genome-wide analyses, have identified a significant negative correlation between tissue-specific promoter methylation and gene expression under physiological conditions (Eckhardt et al., 2006; Gigek et al., 2014; Illingworth et al., 2008; Khulan et al., 2006; Kitamura et al., 2007; Rakyan et al., 2008; Zhang et al., 2013). Variations in DNA methylation have been observed during development and ageing (Xiao et al., 2019). Furthermore, CpG promoter hypermethylation associated with chronological age has also been found to be negatively correlated with gene expression (Zampieri et al., 2015). Regarding histones, modifications of the N-terminal tails of histones within nucleosomes can change the chromatin environment and alter the compactness of the DNA, and hence the recruitment of proteins that modify the accessibility of DNA to transcription factors and the RNA polymerase machinery (Gibney and Nolan, 2010). For example, histone tail modification 3 trimethyl lysine 27 (H3K27me3) in the promoter region is a hallmark of facultative heterochromatin that allows for plasticity in gene expression and variations among different developmental stages and cell types, and between healthy and diseased states of the same organism (Wiles and Selker, 2017). Furthermore, a lack of transcription has been observed to trigger H3K27me3 accumulation in the gene body (Hosogane et al., 2016). In contrast, acetylated (ac) H2A.Z (acetyl K4+K7+K11), when present in the promoter and transcription start site (TSS), is positively correlated with the transcriptional output (Valdés-Mora et al., 2012) and is inversely correlated with promoter H3K27me3 and DNA methylation (Zilberman et al., 2008).

These characteristics of epigenetic modifications led us to hypothesize that this type of modification could be responsible, at least in part, for the modulation of TDP-43 level in mice. The data we present here establish a cause-effect relationship between epigenetic modifications that occur on the promoter and histones of mouse *Tardbp* and the level of TDP-43 both in tissues and in cell culture. Additionally, we also demonstrate that methylation regulates TDP-43 level in a human cell line.

## RESULTS

### Tissue-specific and age-related decay of mouse TDP-43 protein and mRNA levels

**Fig. 1.**
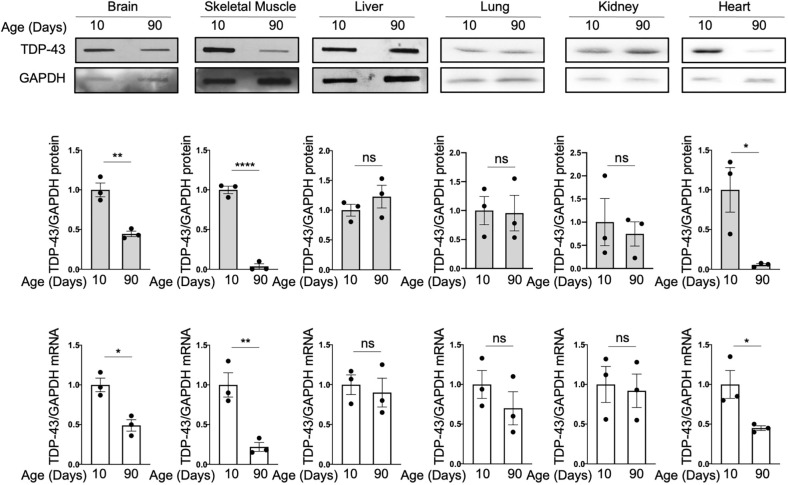
**TDP-43 relative expression levels at 10 and 90 days in different mice tissues.** (A) Upper panel: representative western blots showing TDP-43 levels in 10- and 90-day mice tissues (*n*=3, two males and one female for each group). GAPDH was used as loading control. Middle panel: quantification of the western blots, showing the relative expression level of TDP-43 in the tissue between the two timepoints. Lower panel: quantification of relative TDP-43 mRNA levels between the two timepoints. Values are normalized for GAPDH and expressed as fold over TDP-43 level in 10-day-old tissues. Data are mean±s.e.m. (*n*=3 with two technical replicates for each animal sample). Black circles represent individual data points. Pairwise comparison was performed using an unpaired two-tailed Student's *t*-test. *P<0.05, **P<0.01; ****P<0.0001; ns, not significant.

Following our previous observations regarding the variations in the TDP-43 mRNA and protein levels with increasing age in the *Drosophila* and mouse brains (Cragnaz et al., 2015), we performed an analysis of TDP-43 mRNA and protein expression levels in different tissues of mice aged 10 and 90 days. Our studies showed unequivocally that each tissue has a specific pattern of TDP-43 expression at the two age points studied. It can be seen in Fig. 1 that the levels of TDP-43 decreased significantly not only in brain but also in heart and skeletal muscle. Lung and kidney showed a trend towards lower levels but the differences were not significant. The liver levels, on the other hand, were the same at the two ages analyzed. In all tissues and at all ages, TDP-43 mRNA levels were consistent with the protein levels (Fig. 1). To minimize the possibility that these quantifications were influenced by differential expression of the normalizing protein GAPDH in relation to postnatal development, total protein staining was used to analyze the levels of GAPDH in the brain, skeletal muscle and liver from 10 and 90 days, with no significant difference observed (Fig. S1). Additionally, total protein staining was used to normalize TDP-43 levels in the tissues at the different timepoints and showed analogous results when normalized with GAPDH (Fig. S1). Furthermore, no significant difference in the GAPDH real-time PCR Ct values was observed between the different ages (Fig. S1).

This study was extended to the liver, skeletal muscle and brain tissues of 360-day-old mice (Fig. S2). No further significant reduction in TDP-43 levels in the brain, skeletal muscle and liver were found from day 90 to day 360.

To date, the only known mechanism for modulating cellular levels of TDP-43 consists of an autoregulation loop (Avendaño-Vázquez et al., 2012; Ayala et al., 2011; Bembich et al., 2014; Polymenidou et al., 2011), but this mechanism does not explain the observed reduction in TDP-43 levels in specific tissues and their maintenance at constant levels in other tissues. This fact suggests the existence of an additional mechanism for the modulation of TDP-43 expression, probably at the transcriptional level.

### *Tardbp* promoter methylation shows tissue and time specificity, and inversely correlates with TDP-43 levels

**Fig. 2.**
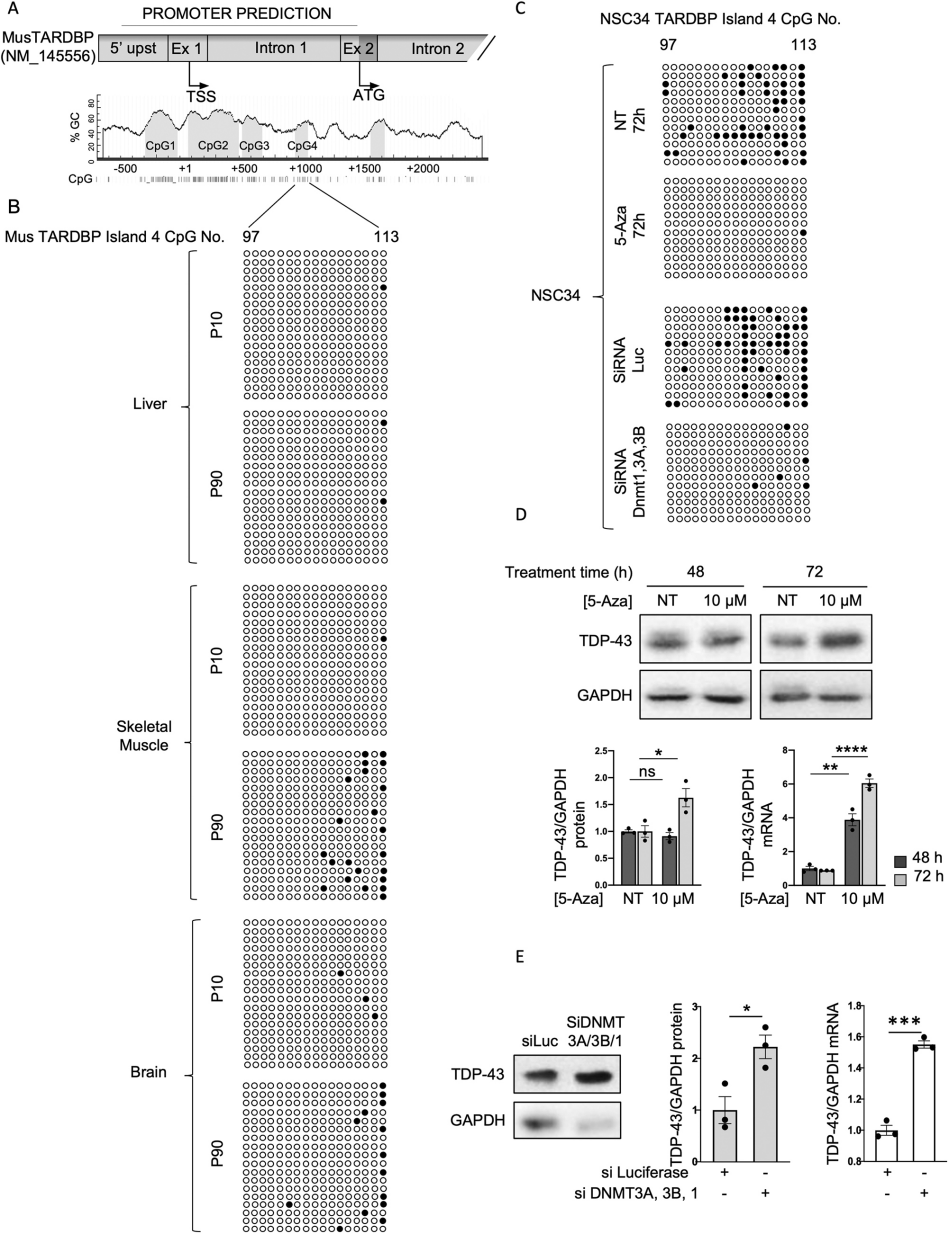
***Tardbp* methylation inversely correlates with TDP-43 expression.** (A) Cartoon of the mouse *Tardbp* promoter predicted to contain 113 CpG sites, encompassing the four CpG islands. Numbering is in respect to TSS. (B) Bisulphite sequencing analysis of the mouse *Tardbp* promoter in the tissues indicated on the left. Each circle represents a CpG site in island four. Dark circles represent sites where DNA was observed to be methylated. Sequencing data came from a first set of six animals (four males and two females). Each line represents a clone (two to four clones for each animal). (C) Bisulphite sequencing analysis of the *Tardbp* promoter in NSC-34 cells before and after 5-AZA treatment, and treated with siRNA against luciferase or siRNA against DNMT1, 3A and 3B. Each circle represents a CpG site in island four. Dark circles represent sites where DNA was observed to be methylated. Each row is an independent clone that was sequenced (*n*=12: four clones sequenced for each of three biological replicates). (D) Upper panel: representative western blots of TDP-43 and GAPDH in NSC-34 cells not treated (NT) and treated with 10 μM of 5-AZA for 48 or 72 h. Bar charts show the quantification after 48 h (dark grey bars) and 72 h (light grey bars) of protein (left) and mRNA (right). Values are normalized for GAPDH and expressed as fold over TDP-43 level in NT cells at 48 h (*n*=3; for qPCR data, two technical replicates for each of three biological replicates were performed). Black circles represent individual data points. (E) Representative western blot of TDP-43 expression before and after knockdown of DNMT3A, 3B and 1 in NSC-34 cells. Bar charts show the quantification of protein (grey) and mRNA (clear). Values are normalized for GAPDH and expressed as fold over TDP-43 levels in control (siLuc; *n*=3; for qPCR data, two technical replicates for each of three biological replicates were performed). Black circles represent individual data points. *P<0.05, **P<0.01; ***P<0.001; ****P<0.0001; ns, not significant (pairwise comparison was performed using an unpaired two-tailed Student's *t*-test).

The characteristics of DNA methylation, described in the introduction, led us to investigate whether this type of modification could be responsible for our observation of tissue- and time-specific TDP-43 levels in mice. For the assessment of DNA promoter methylation, we focused our studies on the brain, skeletal muscle and liver. TDP-43 levels between 10 and 90 days of age both in the brain and muscle exhibited marked decreases in TDP-43 mRNA and protein levels, whereas in the liver, TDP-43 protein and mRNA levels were identical between the two timepoints. Furthermore, the variations in the TDP-43 levels in these tissues are of interest with respect to neurodegenerative pathologies, such as ALS. An *in silico* analysis of the region upstream of the *Tardbp* gene indicated that the putative *Tardbp* promoter sequence consists of a sequence covering positions −562 to 1403 bp relative to the TSS of NM_145556 TDP-43 transcript (according to Eukaryotic Promoter Database, https://epd.epfl.ch//index.php) (Fig. 2A). The MethPrimer prediction software program (Li and Dahiya, 2002) identified 113 CpG sites within this region and clustered these sites into four CpG-rich islands containing the CpG sites numbered 1-28, 29-77, 78-91 and 97-113 (Fig. 2A).

To determine the existence of a correlation between the methylation profile of the murine TDP-43 promoter and the TDP-43 levels, DNA from brain, muscle and liver tissues of mice aged 10 and 90 days was obtained and subjected to bisulphite sequencing (Fig. 2B). Rather than widespread methylation of the CpG islands in the promoter, we found that a specific increase in the methylation of CpG sites 109 through 113 in island 4 was correlated with the decrease in TDP-43 expression observed in skeletal muscle and brain tissues of 90-day-old mice. In the liver, where TDP-43 levels were comparable between 10- and 90-day-old tissues, no increase in methylation was observed.

The transcriptional repression of a promoter mediated by a limited cluster of methylated CpG sites is an uncommon but not unique event (Zou et al., 2006). In fact, even single-site methylation in promoter regions has been shown to repress transcription (Gonzalgo et al., 1998; Pogribny et al., 2000; Robertson et al., 1995). Therefore, the increased methylation of CpG sites 109 through 113 in island 4 might indeed be consistent with a tissue- and age-specific programmed reduction in TDP-43 transcription modulated at least in part by promoter DNA methylation.

### DNA hypomethylation increases TDP-43 expression levels in mouse NSC-34 cells

To substantiate the correlation between methylation and the reduction in TDP-43 levels, we investigated the relationship between *Tardbp* promoter hypermethylation and hypomethylation, and TDP-43 protein expression in mouse motor neuron-derived NSC-34 cells. We initially performed bisulphite sequencing analysis of the four CpG islands in the promoter region to determine the methylation status. Although more widespread and pronounced, a methylation pattern similar to that observed in the mouse tissues was observed in the cultured cells, with the methylation restricted mainly to the fourth CpG island (Fig. 2C). We next evaluated the effects of the demethylation agent 5-azacitydine (5-AZA) (Wozniak et al., 2007) on TDP-43 expression at both the mRNA and protein levels. Treatment with 5-AZA reduced the methylation of the promoter (Fig. 2C), which was associated with a significant increase in TDP-43 mRNA and protein levels in NSC-34 cells (Fig. 2D).

A complementary experiment was performed in which the effect on TDP-43 levels resulting from a reduction in methyltransferase activity in NSC-34 cells was analyzed. DNA methyltransferases (DNMTs) are a family of enzymes that transfer the methyl group from the universal methyl donor S-adenosyl-L-methionine (SAM) to the 5-position of cytosine residues in DNA (Jin and Robertson, 2013). DNMT1 is involved in the maintenance of DNA methylation, and DNMT3A and DNMT3B are regarded as *de novo* methyltransferases. Knockdown of the three DNMTs was performed in the NSC-34 cell line, and the expression of TDP-43 RNA and protein was analyzed. Reduced levels of DNMT1, 3A and 3B (Fig. S3) resulted in demethylation of the promoter (Fig. 2C) and in a significant increase of more than 50% in both TDP-43 mRNA and protein expression compared to the control cells (Fig. 2E), further corroborating the association between reduced methylation and increased expression.

### DNA hypermethylation reduces TDP-43 expression levels in mouse NSC-34 cells

**Fig. 3.**
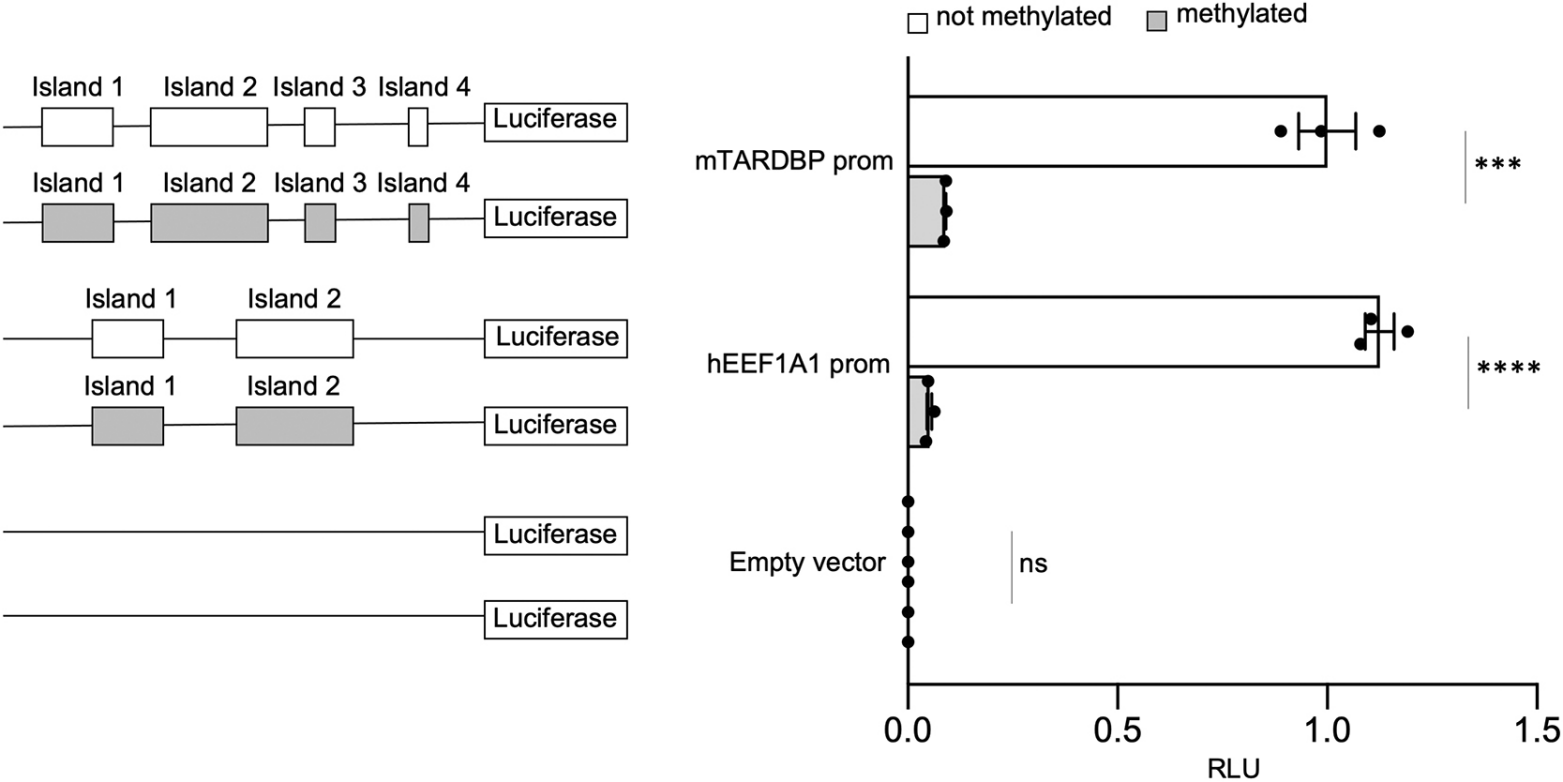
***In vitro* methylation of the mouse *Tardbp* promoter.** EEF1A1 and the empty pCpGfree-basic-Lucia vector were used as positive and negative controls, respectively. On the left of each quantification, cartoons of the constructs used in the assay are shown. White rectangles represent unmethylated CpG islands, and grey rectangles represent methylated CpG islands. Data are mean±s.e.m. (*n*=3). Solid black circles represent individual data points. Pairwise comparison was performed using an unpaired two-tailed Student's *t*-test. ***P<0.001; ****P<0.0001; ns, not significant.

The opposite condition, DNA hypermethylation of the TDP-43 promoter, was then tested. In this assessment, a luciferase reporter assay was performed using the pCpGfree-basic-Lucia construct. A pCpGfree-*Tardbp* construct carrying the promoter region (−562 to +1403 of TSS) identified above was created. *In vitro* methylated or unmethylated plasmids were then co-transfected into NSC-34 cells together with the control vector pGL3, which was used for normalization of transfection efficiency. The promoter activity was evaluated by assessing the ratio of relative luminescence units (RLUs) to pGL3. As a positive control, a vector containing the *EEF1A1* promoter, which is known to be regulated through methylation (Uchiyama et al., 2014), was used, and as expected, this vector was markedly affected by *in vitro* DNA methylation. The results showed that the unmethylated pCpGfree-*Tardbp* construct exhibits significantly higher promoter activity than the methylated construct (>10-fold; Fig. 3).

### Histone modifications associated with transcriptional activity and repression are correlated with *Tardbp* levels

**Fig. 4.**
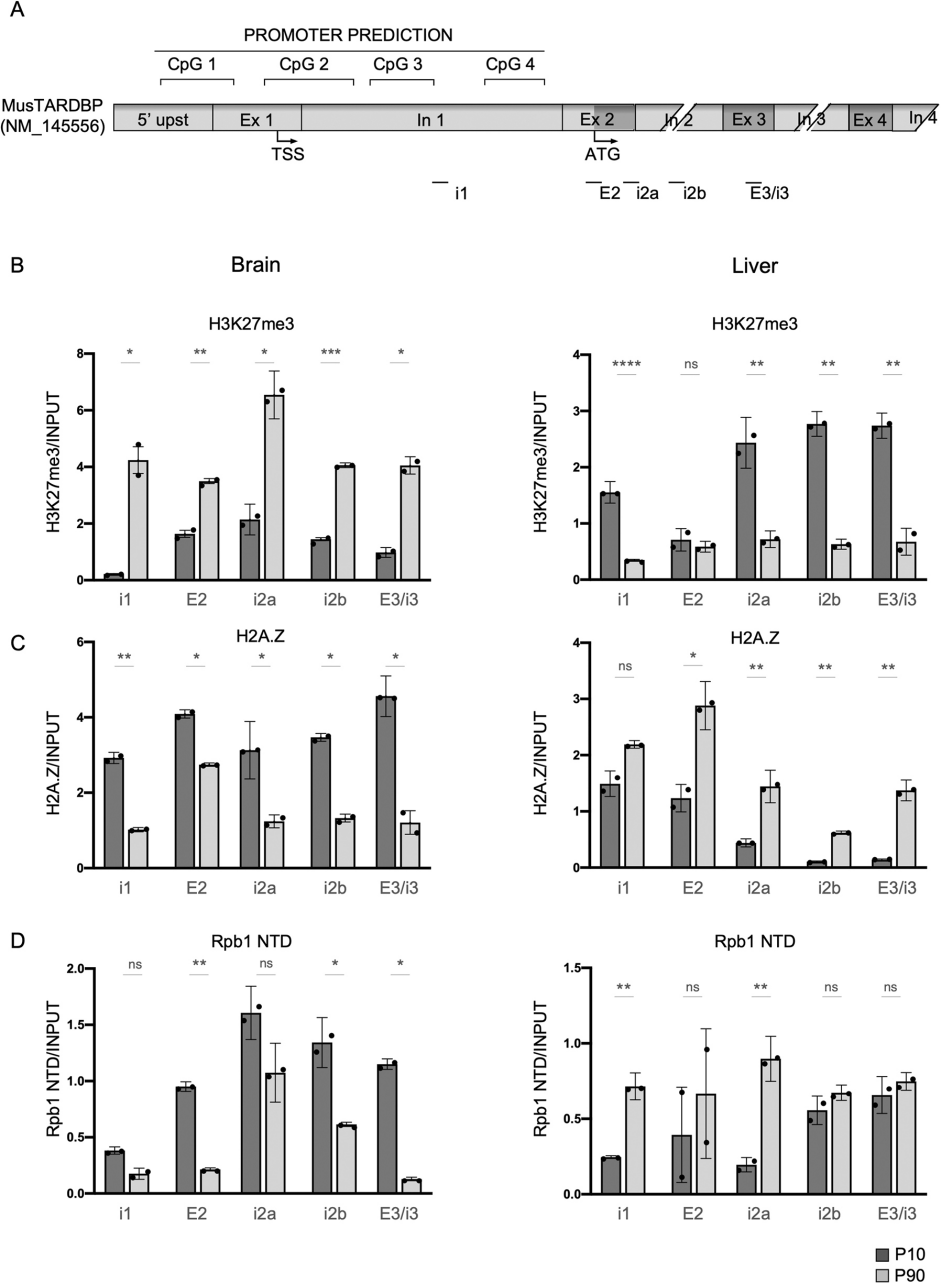
**Analysis of the histone tail modifications, H3K27me3 and acetylated H2A.Z.** (A) Cartoon of the mouse *Tardbp* promoter and the first four intronic/exonic sequences. Regions used for ChIP-qPCR analysis are indicated below. (B-D) Distribution in the promoter and along the gene of K27me3 (B), H2A.Z (C) and RNAPII (Rpb1 NTD) (D) in brain or liver tissue of 10-day-old mice (dark grey columns) compared to 90-day-old mice (light grey columns). Data are mean±s.e.m. (*n*=2 with two technical replicates for each biological replicate). Black circles represent individual data points. Pairwise comparison was performed using an unpaired two-tailed Student's *t*-test. *P<0.05; **P<0.01; ***P<0.001; ****P<0.0001; ns, not significant.

To further investigate epigenetic modifications and their eventual correlation with the age- and tissue-dependent reduction in TDP-43 protein levels, we extended our analysis to representative chromatin modifications preferentially associated with either transcriptionally active or repressed genes, and analyzed these modifications in the promoter-intron 1 region containing the four CpG islands, as well as along the gene body (Fig. 4A). We selected histone tail modification H3K27me3 and histone modification (ac) H2A.Z. As outlined in the Introduction, H3K27me3 is associated with gene repression in the promoter region, as well as along the gene body. (ac) H2A.Z is inversely correlated with promoter H3K27me3 and DNA methylation, and positively correlated with transcription in the promoter region. Little information is available about how acetylation of the variant along the gene body affects gene transcription. Finally, because the recruitment and pausing of RNA polymerase II (RNAPII) in the promoter of actively transcribed genes occurs prior to transcription initiation (Nojima et al., 2015), we also quantified the level of RNAPII in the promoter region and along the gene.

The analysis was performed using chromatin immunoprecipitation (ChIP) with ChIP-grade specific antibodies, which were tested for efficiency, and brain and liver tissues of 10- and 90-day-old mice (Fig. S4), followed by qPCR amplification with relevant primers (Fig. 4A). Accordingly, with the observation of methylation of the promoter and decreased TDP-43 expression in the 90-day-old mouse brain, we found a 4-fold increase in H3K27me3 (Fig. 4B) and a 1.8-fold decrease in acH2A.Z (Fig. 4C) in the brain tissue of these mice. These results are in line with the correlations between these markers described above. Although not significant, a 2.4-fold decrease in RNAPII level was also observed in the promoter region (Fig. 4D). The patterns found along the *Tardbp* gene body in brain tissue were similar to those found in the promoter area and significant in regions E2, i2b and E3/i3. All of these observations are in line with the more open chromatin configuration observed at 10 days and are consistent with the expectations for an actively transcribed gene.

In the liver, which showed constant DNA methylation and TDP-43 levels between the two timepoints analyzed, the pattern of histone modifications found at 10 and 90 days showed a decrease in histone methylation (Fig. 4B) and an increase in acH2A.Z in the promoter region (Fig. 4C), which is indicative of a more open chromatin configuration. RNAPII levels were significantly increased in the promoter and along the gene, except at position E2, i2b and E3/i3 (Fig. 4D). We are currently unable to explain why these markers do not translate to increased levels of TDP-43, and possible explanations are that DNA methylation is dominant in this context and that the autoregulation of TDP-43 at this level of expression maintains equal levels at both ages.

### The age- and tissue-specific variation of TDP-43 levels during development is shared by other RNA-binding proteins

**Fig. 5.**
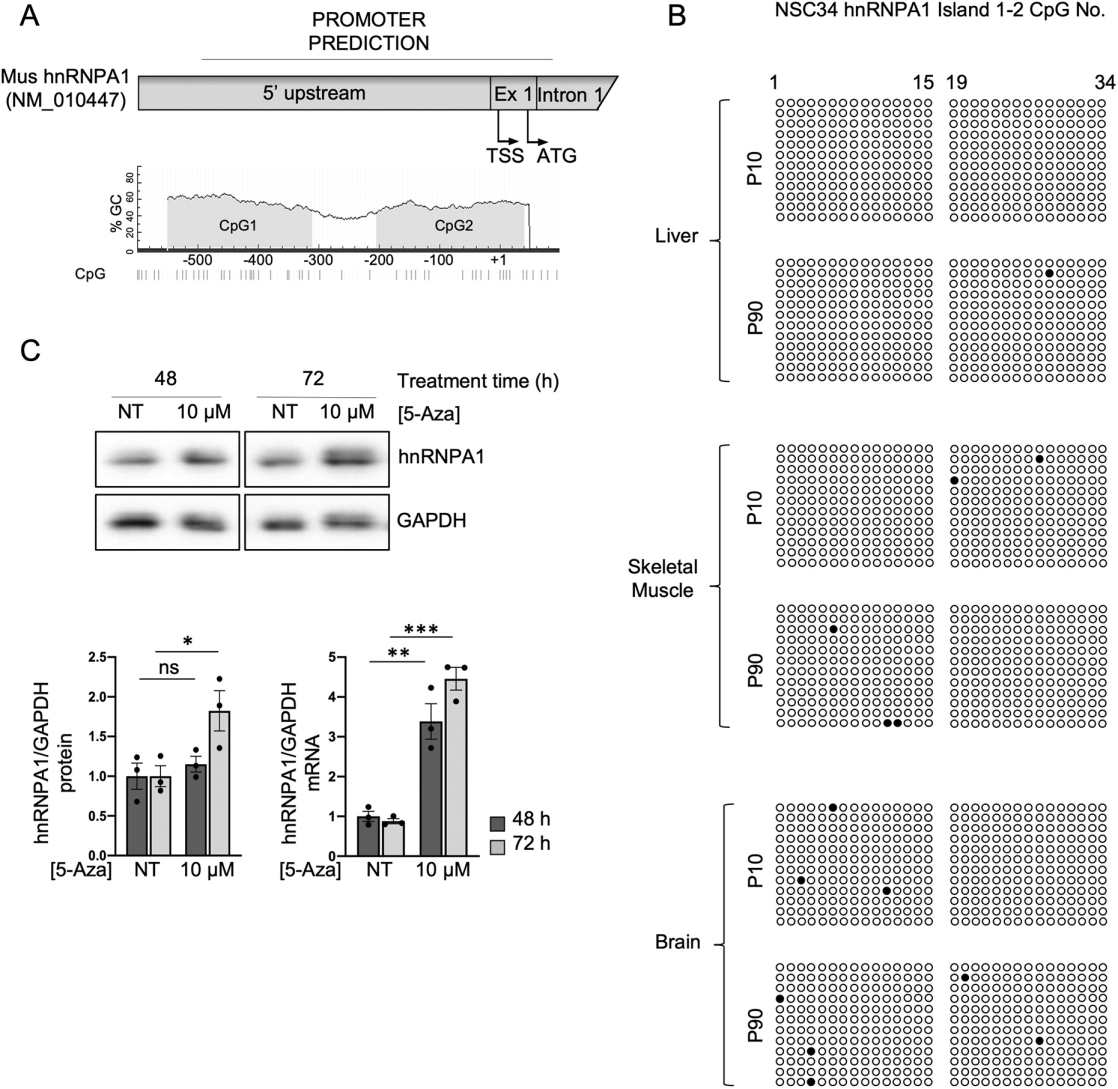
**Methylation analysis of the hnRNPA1 promoter and effect of demethylation by 5-AZA.** (A) Cartoon of the hnRNPA1 promoter predicted to contain two CpG islands. Numbering is in respect to the TSS. (B) Bisulphite sequencing analysis of the mouse hnRNPA1 promoter in the tissues indicated on the left. Each circle represents a CpG site in island one and two. Dark circles represent sites where DNA was observed to be methylated. Sequencing data came from three animals (two males and one female), and each line represents a clone (four clones for each animal). (C) Upper panel: representative western blots of hnRNPA1 and GAPDH in NSC-34 cells not treated (NT) and treated with 10 μM of 5-AZA for 48 h or 72 h. Bar charts show the quantification after 48 h (dark grey bars) and 72 h (light grey bars) of protein (left) and mRNA (right). Values are normalized for GAPDH and expressed as fold over TDP-43 level in NT cells at 48 h (*n*=3; for qPCR data, two technical replicates for each of three biological replicates were performed). Black circles represent individual data points. Pairwise comparison was performed using an unpaired two-tailed Student's *t*-test. *P<0.05; **P<0.01; ***P<0.001; ns, not significant.

Following our observations that epigenetic mechanisms are partly involved in the programmed reduction of TDP-43 levels and given that this protein is a member of the hnRNP family, the same type of modulation might occur with other RBPs. Indeed, previous studies have shown that the expression of various splicing factors changes with ageing (Welle et al., 2003, 2004). Therefore, we quantified several hnRNPs and serine-arginine-rich (SR) proteins in the brain, liver and skeletal muscle (Fig. S5). Similar to TDP-43, hnRNPQ, hnRNPR and SR75 decreased between 10 and 90 days in both the brain and skeletal muscle but exhibited constant levels in the liver. In contrast, the protein levels of hnRNPA1, hnRNPH1 and hnRNPL decreased in all three tissues between the 10- and 90-day timepoint. snRNP70 and hnRNPI also decreased in all three tissues between 10 to 90 days, but significant decreases were found in only the liver and skeletal muscle. To exclude an age-related generalized loss of efficiency in protein expression, particularly in the brain and skeletal muscle, we also quantified the levels of proteins involved in cellular pathways other than RNA metabolism (Fig. S6). The GSK3β protein levels remained constant over the two timepoints in all the tissues, whereas the SOD1 protein levels remained constant in the brain and skeletal muscle, and increased in the liver, which indicates that the differences observed with the splicing factors are not casual. To investigate whether epigenetic control might also play a role in regulating the expression levels of at least some of these factors, we selected hnRNPA1, which decreased in the brain, skeletal muscle and liver between 10 and 90 days, as a protein that exhibited a difference from TDP-43. An *in silico* analysis of the hnRNPA1 promoter was performed and identified a sequence spanning from −499 bp to 101 bp relative to the TSS (NM_010447) according to the Eukaryotic Promoter Database, which contained two CpG islands, as the promoter (Fig. 5A). We then analyzed hnRNPA1 promoter methylation in the three tissues at day 10 and day 90 by bisulphite sequencing, as well as the effects of the demethylation agent 5-AZA on mRNA and protein levels in NSC-34 cells. Bisulphite sequencing of the two CpG islands in 10- and 90-day-old tissue showed the islands were not methylated either at day 10 or day 90 (Fig. 5B). These observations strongly suggest that other molecular mechanism are responsible for the decrease in hnRNPA1 protein levels in tissues between 10 and 90 days, notwithstanding that treatment of the NSC-34 cells with the demethylation agent 5-AZA resulted in a significant increase in protein and mRNA levels (Fig. 5C).

### *In vitro* hypomethylation and hypermethylation are associated with increased and decreased levels of TDP-43, respectively, in human SH-SY5Y cells

RNA-seq data from human brains (Colantuoni et al., 2011) have revealed that TDP-43 exhibits an age-related decrease up to 20 years of age and, interestingly, substantial heterogeneity in its levels at later ages, as shown by the data obtained from the study and elaborated in Fig. S7. Because our data show that TDP-43 is at least partially controlled by epigenetic mechanisms in mice, we hypothesized that this form of regulation might also occur in humans. Given the influence of environmental and genetic factors on epigenetic modifications (Sun, 2014), the heterogeneity of the levels observed in the human population during ageing (Fig. S7) can be explained by the diverse environmental conditions that individual humans live in and by the heterogeneity of the genetic background of humans (Reynolds et al., 2020) compared with that of inbred mice.

**Fig. 6.**
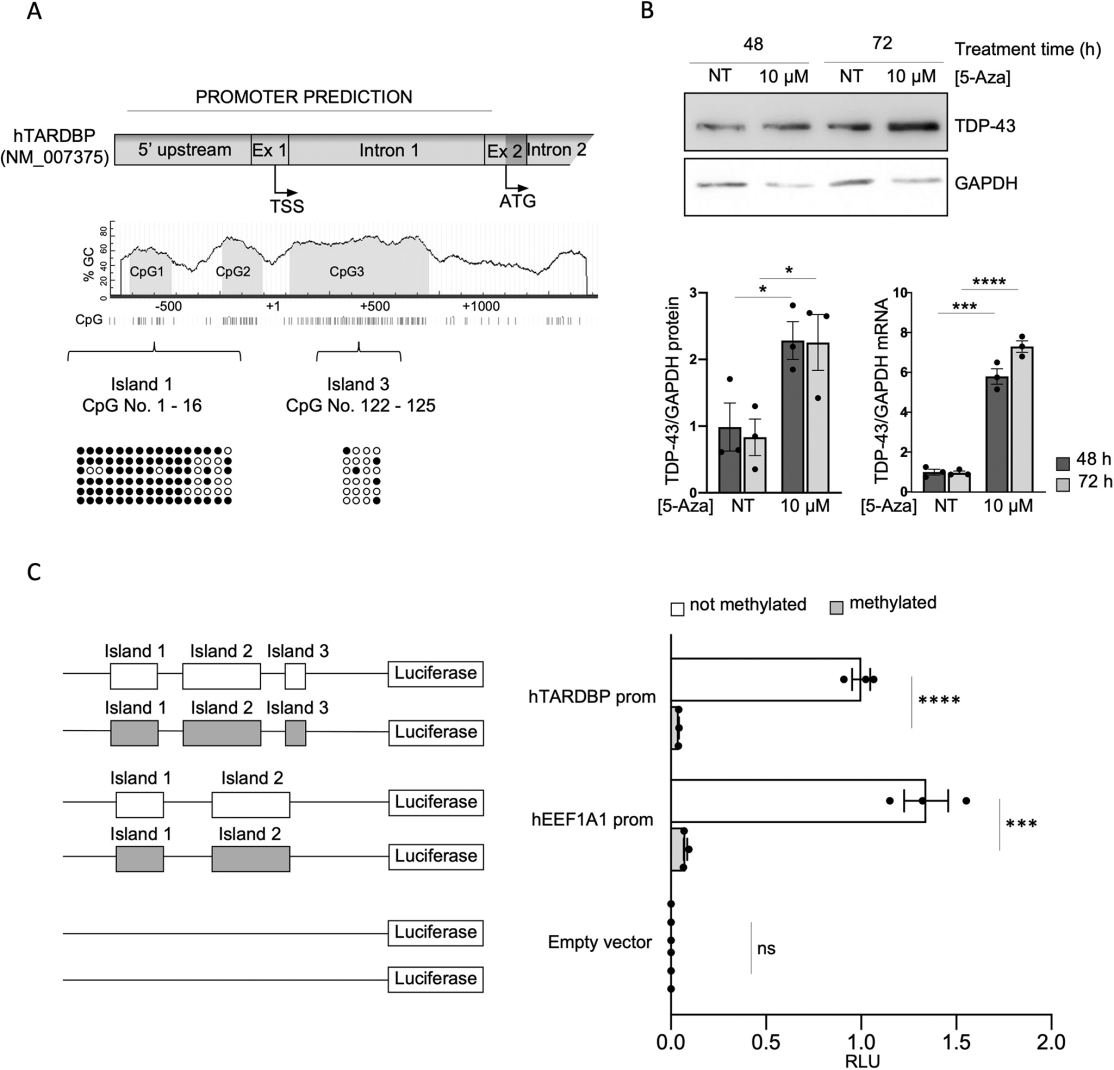
***TARDBP* methylation inversely correlates with human TDP-43 expression.** (A) Cartoon of human *TARDBP* promoter predicted to contain 125 CpG sites, encompassed in three CpG islands. Numbering is in respect to the TSS. Bisulphite sequencing analysis of the promoter in SH-SY5Y cells showed the promoter to be strongly methylated in island one. Each circle represents a CpG site in island one or three. Solid dark circles represent sites where DNA was observed to be methylated. Each line represents a clone (six clones were sequenced). (B) Upper panel: representative western blots of TDP-43 and GAPDH in SH-SY5Y cells not treated (NT) and treated with 10 μM of 5-AZA for 48 or 72 h. Bar charts show the quantification after 48 h (dark grey bars) and 72 h (light grey bars) of protein (left) and mRNA (right). Values are normalized for GAPDH and expressed as fold over TDP-43 level in NT cells at 48 h (*n*=3; for qPCR data, two technical replicates for each of three biological replicates were performed). Black circles represent individual data points. (C) *In vitro* methylation assay of the human *TARDBP* promoter. EEF1A1 and the empty pCpGfree-basic-Lucia vector were used as positive and negative controls, respectively. On the left of each quantification, cartoons of the constructs used in the assay are shown. White rectangles represent unmethylated CpG islands, and grey rectangles represent methylated CpG islands. Data are mean±s.e.m. (*n*=3). Black circles represent individual data points. Pairwise comparison was performed using an unpaired two-tailed Student's *t*-test. *P<0.05; ***P<0.001; ****P<0.0001 ns, not significant.

An *in silico* analysis of the human *TARDBP* promoter was performed using promoter prediction tools. As in the analysis of the mouse promoter, we selected the largest region predicted from all the tools used, which consisted of a genomic area spanning 874 bp upstream to 1069 bp downstream of the TSS (NM_007375, according to Eukaryotic Promoter Database; Fig. 6A). Alignment of the human, mouse and rat putative *TARDBP* promoter sequences has been previously shown to have a low degree of global similarity, and in fact using MethPrimer software (Li and Dahiya, 2002), a different arrangement and number of CpG sites were identified. In the human *TARDBP* promoter, 125 CpG sites within this region were identified, and these could be mainly grouped into three CpG islands (Fig. 6A). Bisulphite sequencing of the region in SH-SY5Y cells showed that the sites in the most upstream island were methylated, whereas sparse methylation was seen in the other islands (Fig. 6A). This finding was different from the results obtained with the mouse *Tardbp* promoter, which revealed that methylation occurred in the more downstream island. We then tested the relationship between *TARDBP* promoter hypomethylation and TDP-43 expression in human cells by evaluating the effects of the demethylating agent 5-AZA on TDP-43 mRNA and protein levels, and observed that these were significantly increased at 48 and 72 h (Fig. 6B). Hypermethylation of the promoter was also tested using the pCpGfree-basic-Lucia construct and by inserting the promoter region described above. The promoter activity was evaluated by assessing the ratio of RLUs to pGL3 (Fig. 6C). The results showed that the unmethylated pCpGfree-*TARDBP* construct exhibited significantly higher promoter activity than the methylated construct (>10-fold). A vector containing the *EEF1A1* promoter, which is known to be regulated by methylation, was used as a positive control, and none of the constructs analyzed were affected by any residual activity of the pCpGfree backbone of the vector, which did not show any significant variation when methylated *in vitro*.

## DISCUSSION

The central role of RBPs in the regulation of RNA metabolism and gene expression implies tight control of the regulation of RBP proteostasis during development and adulthood. This regulation is achieved via several different mechanisms (Dassi, 2017; Risso et al., 2012) for a specific RBP, as well as in a coordinated manner between members of this family (Quattrone and Dassi, 2019), and this coordination results in tissue splicing networks (Baralle and Giudice, 2017). Alternative splicing is one mechanism through which specific RBPs control the expression of other RBPs. These alternative splicing events create RNAs with premature stop codons that, coupled with nonsense-mediated decay, negatively regulate gene expression post-transcriptionally (Ni et al., 2007). In addition, tissue-specific and/or temporally regulated expression of miRNAs modulate the levels of several RBPs (Boutz et al., 2007; Meseguer et al., 2011; Song et al., 2017). Moreover, another major mechanism responsible for the regulation of RBP mRNA levels involves an autoregulatory feedback loop that serves to maintain protein abundance within a physiological range. This mechanism has been reported for a number of members of this family, including TDP-43 (Müller-McNicoll et al., 2019).

To the best of our knowledge, the role of epigenetic modifications in the physiological gene expression of RBPs throughout the lifespan of an organism has not been addressed to a significant extent. However, for example, CELF2, an RBP involved in alternative splicing, has been observed to undergo promoter CpG island hypermethylation-associated transcriptional silencing in humans. The loss of CELF2 is associated with an altered downstream pattern of exon usage in several target genes, and this phenomenon has been observed to enhance the growth of breast tumours (Piqué et al., 2019)

Our study provides the first line of evidence showing that epigenetic modifications in the *Tardbp* promoter are responsible for the differential expression of TDP-43 in mouse tissues at two different ages, and the results indicate the existence of additional regulatory mechanisms. The existence of a physiological evolutionarily conserved programme for the epigenetic regulation of TDP-43 and potentially other RBPs has wide implications, and might help us to understand processes such as ageing and the pathological degeneration of specific tissues.

Given the role of TDP-43 in ALS, whether the reduction in TDP-43 level during ageing occurs in human motor neurons in the same manner as in mice is of extreme interest. Unfortunately, this mechanism remains to be established. However, the available whole-brain RNA-seq data on TDP-43 levels during ageing show large heterogeneity (Fig. S7). Furthermore, our *in vitro* experiments showed that hypermethylation and hypomethylation of the *TARDBP* promoter can substantially affect the levels of mRNA and protein. As variations in DNA methylation have been observed during development and ageing (Xiao et al., 2019), epigenetic changes resulting in chromatin remodelling leading to higher or lower accessibility to the genome might thus play a critical role in modulating the TDP-43 protein levels in an age- and tissue-dependent manner in humans. As a consequence, the heterogeneity of TDP-43 levels observed with ageing in humans could be explained by the wide variety of individual genetic make-ups and living environments of humans, which influence epigenetic modifications to a larger extent than the effects observed in an inbred mouse strain.

**Fig. 7.**
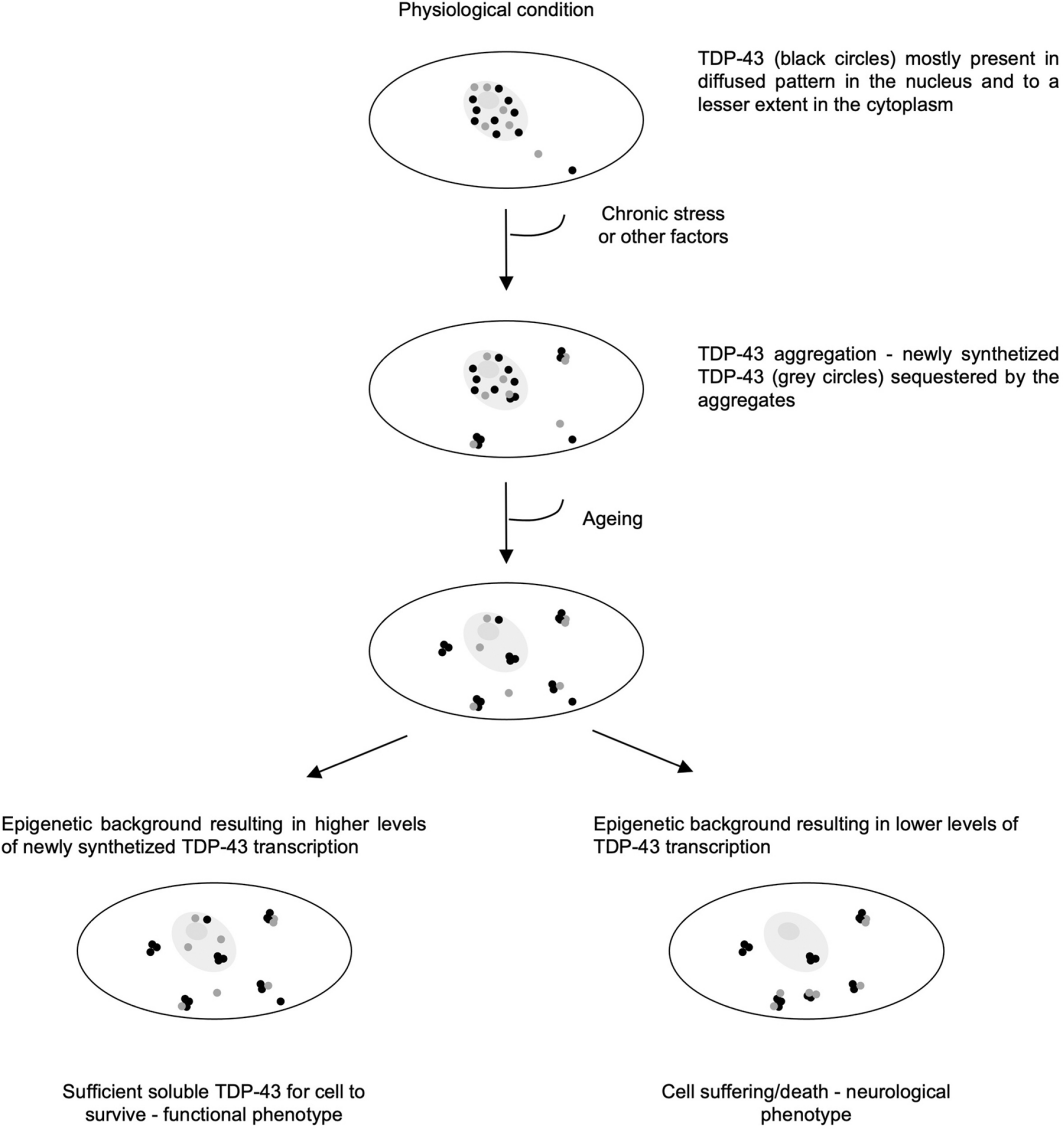
**Model of possible epigenetic modifications of TDP-43 levels and their contribution to ALS.** TDP-43 is mostly present in a diffused pattern in the nucleus and to a minor extent in the cytoplasm. When the normal balance is perturbed either because of prolonged stress conditions, impairment of particular pathways or modifications of TDP-43 protein structure, the tendency of TDP-43 to aggregate is enhanced. If the epigenetic makeup of the *TARDBP* promoter is such that high levels of the protein are transcribed (left panel), even in the presence of aggregation, the cell may still have a functional phenotype. If the epigenetic makeup of the *TARDBP* promoter is such that the levels are low, the cell will eventually die (right panel).

Whether TDP-43 aggregates trigger a gain- or loss-of-function effect, or eventually a combination of these two effects, remains an unanswered question regarding the pathogenesis of ALS. However, the genetic suppression of TBPH in flies (Feiguin et al., 2009) has been shown to result in an ALS-like phenotype, and a similar finding has been observed after ablation of a single *Tardbp* allele in heterozygous transgenic mice (Kraemer et al., 2010). In fact, in this study, the lack of compensation for the reduction in TDP-43 levels reinforces the notion of overlapping regulatory mechanisms, which indicates that transcription is fixed at a given age because the levels could be compensated by the other allele only if autoregulation was active. In any case, the decrease in TDP-43 associated with ALS-like pathology is consistent with the model that the pathogenesis is substantially driven by loss of TDP-43 function (Vanden Broeck et al., 2014). These observations, together with the fact that 29% of healthy individuals over 65 years of age present TDP-43 inclusions in the brain but no pathology, suggest that heterogeneous levels of human wild-type TDP-43 could play a fundamental role in the pathogenesis (Uchino et al., 2015). This finding is further corroborated by the fact that in a transgenic fly constitutively expressing a TDP-43 aggregation inducer (ALS-like model), the main physiological decrease in TDP-43 during fly ageing (∼4-fold at 10 days) coincides with the onset of the locomotion defect in this model. A transgenic fly with a constitutively low level of TBPH from birth exhibits an earlier onset of the pathological phenotype (Cragnaz et al., 2015). These observations link the combination of TDP-43 aggregation and age-related physiologically low levels of TDP-43 with ALS-like pathology. It is plausible that individuals showing reduced TDP-43 levels in the brain and the presence of pathological aggregates exhibit a predisposition to ALS. ALS is a highly heterogeneous disease, as reflected by variations in the age of onset, site of onset, disease progression rate and survival. In fact, the precise mechanisms of pathogenesis remains to be elucidated. It is tempting to hypothesize that the physiological variability in the levels of TDP-43 observed to date might play a role in the onset of ALS. For example, as shown in Fig. 7, the continuous capture of newly synthesized TDP-43 by inclusions, which has been observed in most ALS patients (Neumann et al., 2006), will only be pathological if the age-related reduction in TDP-43 is equal to or less than the capacity of the inclusion to capture this protein, which results in loss of its nuclear function. The heterogeneity in humans, which is due at least in part to different epigenetic modifications, will result in different TDP-43 levels and hence differences in the ability to compensate for the loss of TDP43 due to sequestration by aggregates.

Furthermore, characterizing the epigenetic control of TDP-43 protein levels and understanding the complexity of the epigenetic modulation of TDP-43 expression might be considered a first step toward identifying a new potential mechanism for restoring TDP-43 functionality, which is lost in ALS-affected tissues. Interestingly, several studies have shown that whole-global DNA methylation is increased in ALS (Coppedè et al., 2018; Hamzeiy et al., 2018; Tremolizzo et al., 2014) and that some members of the hnRNP family ameliorate or worsen neurodegeneration (Appocher et al., 2017). Moreover, epigenetic regulation might be more widespread in the RBP family, as indicated by our observation of a tissue-specific decrease in the levels of several members of this protein family in mice. Although our analysis of the hnRNPA1 promoter did not show promoter methylation in the tissues associated with the decrease between 10 and 90 days, the demethylation agent 5-AZA did increase hnRNPA1 protein and mRNA levels, possibly indicating that the epigenetic control is not coming from the promoter. The impact of RBP level modulation on several physiological and pathological processes extends beyond neurodegenerative diseases, and many splicing defects might be either accentuated or attenuated by this phenomenon. These findings uncover new potential targets for the modulation and therapy of splicing deficiencies, which might have a wide-ranging impact in a substantial range of pathologies in which the subtle effect of splicing variations resulting from RBP-level changes might pass unnoticed yet could be the main cause of the disease.

## MATERIALS AND METHODS

### Cell culture and gene knockdown

NSC-34 and SH-SY5Y cell lines were obtained from the American Type Culture Collection (ATCC Microbiology, Manassas, VA, USA) and were cultured in Dulbecco's modified Eagle's medium-Glutamax-I (Gibco-BRL, Life Technologies, Frederick, MD, USA,) supplemented with 5% fetal bovine serum (Sigma-Aldrich, St Louis, MO, USA) and 10% fetal bovine serum (Gibco-BRL, Life Technologies Inc), and 1% antibiotic-antimycotic solution (Sigma-Aldrich), in a 37°C incubator with a humidified atmosphere of 5% CO_2_. Tissue cultures were routinely tested for mycoplasma contamination every 3 months using a MycoAlert Mycoplasma Detection Kit (LT07-218).

NSC-34 cells were co-transfected twice in two consecutive days with small interfering (si)RNAs targeting DNMT1 (FlexiTube GeneSolution, GS13433), DNMT3A (Sigma-Aldrich, target sequence 5′-ACUCUAUAAAGCAGGGCAA-3′) and DNMT3B (FlexiTube GeneSolution GS13436) using Lipofectamine RNAiMAX Transfection Reagent (Thermo Fisher Scientific) according to the manufacturer's instructions. On the third day, cells were harvested, divided into two aliquots and processed for protein and RNA analyses.

### Animal tissue samples

Male/female FVB/N wild-type mice (Charles River) aged 10 and 90 days were sacrificed, and tissues were surgically removed, frozen in dry ice and stored in a freezer at −80°C until analysis. Before protein, RNA and DNA extractions, tissues were pulverized in liquid nitrogen and divided into three homogeneous aliquots. Animal procedures were approved by the ICGEB Animal Ethics committee. This committee works according to the Italian law D.L.vo n.26/2014, which establishes and regulates animal experimentation.

### Total RNA extraction from cells and mouse tissues, and real-time PCR

RNA from cell cultures was extracted using Eurogold TriFast (EuroClone, Milan, Italy), following the manufacturer's instructions. RNA from tissues was extracted following the same procedure upon pulverization in liquid nitrogen and homogenization in Eurogold TriFast using a mechanical agitator (ForLab, Bergamo, Italy). RNA (1 μg) was reverse-transcribed using Moloney murine leukemia virus reverse transcriptase (Invitrogen, Carlsbad, CA, USA) and random/oligo-dT primers (Sigma-Aldrich) according to the manufacturer's instructions. Total cDNA was used to perform qPCR using the following primers: qPCR musTDP-43 Fw, 5′-GCAGTCCAGAAAACATCTGACC-3′ and qPCR musTDP-43 Rv, 5′-ACACCATCGCCCATCTATCAT-3′; qPCR musGAPDH Fw, 5′-AGGTCGGTGTGAACGGATTTG-3′ and qPCR musGAPDH Rv, 5′-TGTAGACCATGTAGTTGAGGTCA-3′; qPCR hTDP-43 Fw, 5′-ATCTGGTGTATGTTGTCAACTATCC-3′ and qPCR hTDP-43 Rv, 5′-GAACTTCTCCAAAGGTACTAAAATACTC-3′; qPCR hGAPDH Fw, 5′-AAGGTGAAGGTCGGAGTCAA-3′ and qPCR hGAPDH Rv, 5′-AATGAAGGGGTCATTGATGG-3′, and qPCR mushnRNPA1 Fw, 5′-GTCCGAGTCTCCCAAGGAGCC-3′ and qPCR mushnRNAPA1 Rv, 5′-TGACAAACCCAAAGCCCCTGG-3′. qPCR was performed using iQ SYBR Green Supermix (Bio-Rad, Hercules, CA, USA) and a C1000 Thermal Cycler CFX96 Real Time System (Bio-Rad). Expression of the gene of interest was normalized to the GAPDH housekeeping gene. Data were analyzed using the 2^−ΔΔCt^ method (Schmittgen and Livak, 2008). The mean of relative expression levels±s.e.m. of three independent experiments is reported. Pairwise comparison between groups was performed using Student's *t*-test. *P*<0.05 was considered statistically significant.

### Inhibition of DNA methylation

NSC-34 and SH-SY5Y cells were treated with 10 μM of the DNA methyltransferase inhibitor 5-AZA (Sigma-Aldrich) for 48 and 72 h. Medium with 5-AZA was renewed every 24 h. Cells treated with H_2_O, the vehicle for 5-AZA suspension, were used as control. After 48 or 72 h, cells were harvested into three aliquots for RNA and protein and genomic DNA extraction.

### Immunoblotting

Total cell protein extracts were obtained from lysis in 15 mM HEPES (pH 7.5), 250 mM NaCl, 0.5% (v/v) NP-40, 10% (v/v) glycerol and 1× protease inhibitors (Roche), and sonication for 5 min at the highest potency. Protein extracts from mouse tissues were obtained by pulverizing the tissue in liquid nitrogen and homogenizing in lysis buffer using a mechanical agitator (ForLab). Tissue lysates were cleared of debris by centrifugation at 8000 ***g*** for 5 min. Total cell proteins (20 μg) or mouse tissue proteins (50 μg) were separated using SDS-PAGE, blotted onto a nitrocellulose membrane and probed with the following primary antibodies: anti-TDP43 (Proteintech, 10782, 1:1000); anti-hnRNPA1 (Proteintech, 11176, 1:1000); anti-hnRNPH1 (in house, 1:1000); anti-hnRNPI (PTB) (in house, 1:1000); anti-hnRNPL (Abcam, ab6106, 1:2000); anti-hnRNPQ (Sigma-Aldrich, HPA041275, 1:1000); anti-hnRNPR (Abcam, ab30930, 1:1000); anti-snRNP70 (Abcam, ab83306, 1:1000); anti-1H4 (SR75) (Merck, MABE50, 1:1000); anti-GAPDH (Abcam, ab9485, 1:2500), anti-DNMT1 (Abcam, ab19905, 1:1000), anti-DNMT3A (Santa Cruz Biotechnology, sc-20703, 1:1000), anti-DNMT3B (Abcam, ab16049, 1:1000); anti-SOD1 (Abcam, ab16831, 1:2000); and anti GSK-3β (Cell Signaling Technology, 9315, 1:1000). Protein signatures were detected using ECL Western Blotting Substrate (Thermo Scientific, 34106) upon incubation with the following secondary antibodies: horseradish peroxidase (HRP)-labelled anti-mouse (Thermo Scientific, 32430, 1:2000) and HRP-labelled anti-rabbit (Thermo Scientific, 32460, 1:2000). Antibody validation was based on observed molecular weight, which was in accordance with the manufacturer's reported protein signature.

Protein bands were quantified using ImageJ. The intensities of the bands of interest were normalized using the housekeeping gene GAPDH (Abcam, ab9485), or total protein signatures were stained using Ponceau S solution (Sigma-Aldrich, P7170). For the latter, all protein bands per lane were used for ImageJ quantification. Values are presented as a mean of three independent experiments, and error bars indicate s.e.m. Pairwise comparison between groups was performed using Student's *t*-test. *P*<0.05 was considered statistically significant.

### Chromatin immunoprecipitation

Mice tissues were pulverized in liquid nitrogen, washed in PBS and treated for 10 min in ice-cold 1% (v/v) formaldehyde/PBS to crosslink protein-DNA complexes. Crosslinking was stopped with glycine at a final concentration of 125 mM. Samples were homogenized with a Dounce homogenizer and then pelleted by centrifugation at 17,000 ***g*** for 5 min at 4°C, washed in PBS twice and then redissolved in Buffer A [100 mM Tris, 100 mM NaCl, 30 mM MgCl_2_, 0.1% NP-40 and 0.1 mM phenylmethylsulfonyl fluoride (PMSF)]. Samples were homogenized with a Dounce homogenizer again, pelleted and dissolved in Buffer B [1% SDS, 10 mM EDTA, 50 mM Tris-HCl (pH 8) and 0.1 mM PMSF], and then DNA was sonicated to an average length of 200-500 bp. After addition of 1% (v/v) Triton X-100, samples were centrifuged at 15,000 ***g***. Supernatants were immunoprecipitated overnight with 40 µl of precoated anti-IgG magnetic beads (Dynabeads, M-280, Invitrogen) previously incubated with the antibody of interest for 6 h at 4°C. The antibodies used were as follows: anti-H3 (2 μg, Abcam, ab1791); anti K27me3 (4 μg, Abcam, ab6002); anti H2A.Z (acK4+K7+K11) (4 μg, Abcam, ab18262); and anti Rpb1 NTD (2 μg, Cell Signaling Technology, D8L4Y, 14958). Control immunoprecipitations were performed using rabbit IgG (1 μg, Abcam, ab171870). Beads were washed sequentially for 5 min each in low-salt [20 mM Tris-HCl (pH 8), 150 mM NaCl, 2 mM EDTA, 1% Triton X-100 and 0.1% SDS], high-Salt [20 mM Tris-HCl (pH 8), 500 mM NaCl, 2 mM EDTA, 1% Triton X-100 and 0.1% SDS] and LiCl buffer [10 mM Tris (pH 8.0), 1 mM EDTA, 250 mM LiCl, 1% NP-40 and 1% Na-deoxycholate] for 5 min at 4°C and then twice in 1× TE buffer for 2 min at room temperature. Beads were eluted in 1% SDS and 100 mM NaHCO_3_ buffer for 15 min at 65°C, and crosslinking was reversed for 6 h after the addition of NaCl to a final concentration of 200 mM. Chromatin was precipitated with ethanol overnight, treated with 20 μg of proteinase K and purified by phenol-chloroform extraction. Immunoprecipitated DNA (1.5 μl) and serial dilutions of the 10% input DNA (1:4, 1:20, 1:100 and 1:500) were analyzed by SYBR-Green real-time qPCR. The oligonucleotide 5′ to 3′ sequences used were as follows: m_q_Tdp43_p_fw1, 5′-CTGTTCTGATGTCGGTGTTC-3′ and m_q_Tdp43_p_rv1, 5′-TATAGCCTGGACCTGAATCG-3′; m_q_Tdp43_fw1, 5′-AGAAGATGAGAACGATGAACC-3′ and m_q_Tdp43_rv1, 5′-ACTGGGCTGTAACTGTGG-3′; m_q_Tdp43_fw2, 5′-ATAGCCAGGACTACAACAAAGG-3′ and m_q_Tdp43_rv2, 5′-AGCAATCTACCGAGTTCTAACC-3′; m_q_Tdp43_fw3, 5′-CGTGTATCAGAAGAGGGTTG-3′ and m_q_Tdp43_rv3, 5′-TGGTCTACAGAGTGAGTTCC-3′; m_q_Tdp43_fw4, 5′-TGGAGAGGTTCTTATGGTTCAG-3′ and m_q_Tdp43_rv4, 5′-GGACATATCTGCTACATACACAAG-3′; m_q_A1_p_fw, 5′-AGGGCGAGCAGAAGGTAG-3′ and m_q_A1_p_rv, 5′-GACGGTAGAGTTACGAGAGC-3′; m_q_A1_fw1, 5′-CGCTAAACACTCCCTCCTC-3′ and m_q_A1_rv1, 5′-TGGCTCCTCAGACTCTCG-3′; m_q_A1_fw2, 5′-AGTGCTTCATCCAGTCAGAG-3′ and m_q_A1_rv2, 5′-GACCACCACCAAAGTTTCC-3′; m_q_A1_fw3, 5′-ACTTGTATTCTTATAGGTGGTTATGG-3′ and m_q_A1_rv3, 5′-GCCTCCTCCGTTGTTATAGC-3′; and m_q_Gapdh_fw1, 5′-TTAGGTTCATCAGGTAAACTCAG-3′ and m_q_Gapdh_rv1, 5′-GCTACGCCATAGGTCAGG-3′.

### *In vitro* methylated reporter assay

The construct m*Tardbp* prom was created by amplifying a 1965-bp *Tardbp* mouse promoter region (−562 to +1403 of TSS NM_145556) from mouse genomic DNA using the Fw 5′-CGCGGATCCAGAGAGAGTGCTAAAAAGGTACTTGAT-3′ and Rv 5′-CGGGGTACCCTTTGCTTAAATCTCTTAAAGG-3′ primers carrying BamHI and KpnI restriction sites, respectively. The amplicon was subsequently inserted into the CpG-free and promoterless vector pCpGfree-basic-Lucia (InvivoGen, San Diego, CA, USA) upstream of the luciferase reporter gene upon introduction of a KpnI restriction site using the quick-change PCR mutagenesis technique.

The construct hTARDBP prom was obtained by amplifying a 1943-bp *TARDBP* human promoter region [spanning 874 bp upstream and 1069 bp downstream of the TSS (NM_007375)] using human genomic DNA through the forward 5′-GTCTACCATTTATTCCTTG-3′ and reverse 5′-CTTTTACTTTTCCTAAAGAG-3′ primers. A second amplification was performed to add the restriction sites HindIII at the 5′ end and KpnI at the 3′ end, using the forward primer 5′-tatataagcttggtctaccattt-3′ and the reverse primer 5′-ATATATGGTACCCTTTTACTTTT-3′. The fragment was then cloned into the pCpGfree-basic-Lucia vector upstream of the luciferase reporter gene. A positive control of the *in vitro* methylation assay was generated by amplifying a 1107-bp fragment of *EEF1A1* human promoter (−234 to +873 of TSS NM_001402) from human genomic DNA, using Fw 5′-CGCGGATCCTCTCGTCATCACTGAGGTGGA-3′ and Rv 5′-CTAGACTAGTCAAGCTGGCCTAACTTCAGTCTC-3′ primers carrying BamHI and SpeI restriction sites, respectively, and subsequently inserting the fragment into the pCpGfree-basic-Lucia vector upstream of the luciferase reporter gene.

Constructs were then methylated *in vitro* using M.SssI CpG methyltransferase (New England BioLabs) according to the manufacturer's instructions. Briefly, 5 µg of plasmid DNA were added to the reaction containing CpG methyltransferase (M.SssI) in the presence of 160 µM SAM (New England Biolabs), and incubated for 4 h at 37°C (SAM was replenished every 2 h). Unmethylated control reaction containing the construct and methyltransferase, but not SAM, was used. Plasmid DNA was then purified using a Promega miniprep kit and quantified using a Nanodrop spectrophotometer. Methylation was confirmed by digestion with the methylation-sensitive restriction enzyme HpaII.

### Transient transfection and luciferase assay

NSC-34 or SH-SY5Y cells were co-transfected with the methylated and unmethylated vectors described above, and pGL3 luciferase reporter vector (Promega), using Lipofectamine 2000 (Invitrogen). A pCpGfree-basic-Lucia vector with *EEF1A1* promoter and pCpGfree-basic-Lucia empty vector were used as positive and negative controls, respectively. The activity of both pCpGfree-basic-Lucia and pGL3 vectors was measured using the Dual-Glo Luciferase Assay System (Promega) according to the manufacturer's instructions. The promoter activity was expressed as a ratio of luminescence normalized to pGL3 activity in each sample.

### DNA methylation status analysis

DNA from cells or solid mouse tissues was extracted using a quick-DNA Midiprep Plus Kit (Zymo Research) according to the manufacturer's instructions. Bisulfite modification of the genomic DNA was performed using an EpiMark Bisulfite Conversion Kit (BioLabs) according to the manufacturer's instructions. Primers used to amplify the mouse-converted genomic DNA were as follows: m*Tardbp* 1st island Fw, 5′-AGAGAGTGTTAAAAGGTATTTGAT-3′ and m*Tardbp* 1st island Rv 5′-TCTAAAAAACTAACAAATAAAAAACA-3′; m*Tardbp* 2nd (a) island Fw, 5′-GTTTTTTAGATTTAGTTGTTTTTATTG-3′ and m*Tardbp* 2nd (a) island Rv, 5′-CTAAACCACTACTAAAAAACACA-3′; m*Tardbp* 2nd (b) island Fw, 5′-TGTGTTTTTTAGTAGTGGTTTAG-3′ and m*Tardbp* 2nd (b) island Rv, 5′-CCCATAATAACAACAATTATTCTC-3′; m*Tardbp* 3rd island Fw, 5′-GTTGTTATTATGGGATTTGGATTTT-3′ and m*Tardbp* 3rd island Rv, 5′-CAATTATCTAACAATTCTAATACACATCC-3*′*; m*Tardbp* 4th island Fw, 5′-TAGAAGAAATTTAATTTAGAATGG-3′ and m*Tardbp* 4th island Rv, 5′-TACTCTTCAAAAAATCCAAAATTC-3′; mhnRNPA1 1st island Fw, 5′-TTGTGTTTGTGTAATTAATTTTGG-3′ and mhnRNPA1 1st island Rv, 5′-CATAACTTTCCCACACCCAAACATTACAAAC-3′; and mhnRNPA1 2nd island Fw, 5′-GTTTGTAATGTTTGGGTGTGGGAAAGTTATG-3′ and mhnRNPA1 2nd island Rv, 5′-AAACTACAAAACACATCCAACTTAC-3′. Sequences of the primers used to amplify the human converted genomic DNA were as follows: h*TARDBP* 1st island Fw, 5′-GGTTTATTATTTATTTTTTGGGTG-3′ and h*TARDBP* 1st island Rv, 5′-CACATATTTAATAACCCAAACAATT-3′; h*TARDBP* 2nd island Fw, 5′-TAAAGTTATTGTTTTTAGGTTTGG-3′ and h*TARDBP* 2nd island Rv, 5′-TAAAATAAAACCAAAACCTAAACC-3′; h*TARDBP* 3rd (a) island Fw, 5′-TAGGTTTGAATTTTTAGGAGGTAG-3′ and h*TARDBP* 3rd (a) island Rv, 5′-AAAACACCAAACCCAAACTAAC-3′; and h*TARDBP* 3rd (b) island Fw, 5′-GTTAGTTTGGGTTTGGTGTTTT-3′ and h*TARDBP* 3rd (b) island Rv, 5′-CTAACTTCCAAAAAAATACTTATC-3′. PCR products were purified, inserted into the pGEM T-easy vector (Promega) and transformed. Twelve to 18 clones for each condition were sequenced using a Eurofins sequencing system, and the methylation status of each CpG site was reported.

## Supplementary Material

Supplementary information
