# Supplementary material for: Physiological tissue-specific and age-related reduction of mouse TDP-43 levels is regulated by epigenetic modifications
Source: Dis Model Mech. 2022 Apr 29;15(4):dmm049032. doi: 10.1242/dmm.049032 (PMC9066495; doi:10.1242/dmm.049032)
Supplement: Supplementary information [file dmm-15-049032-s1.pdf]

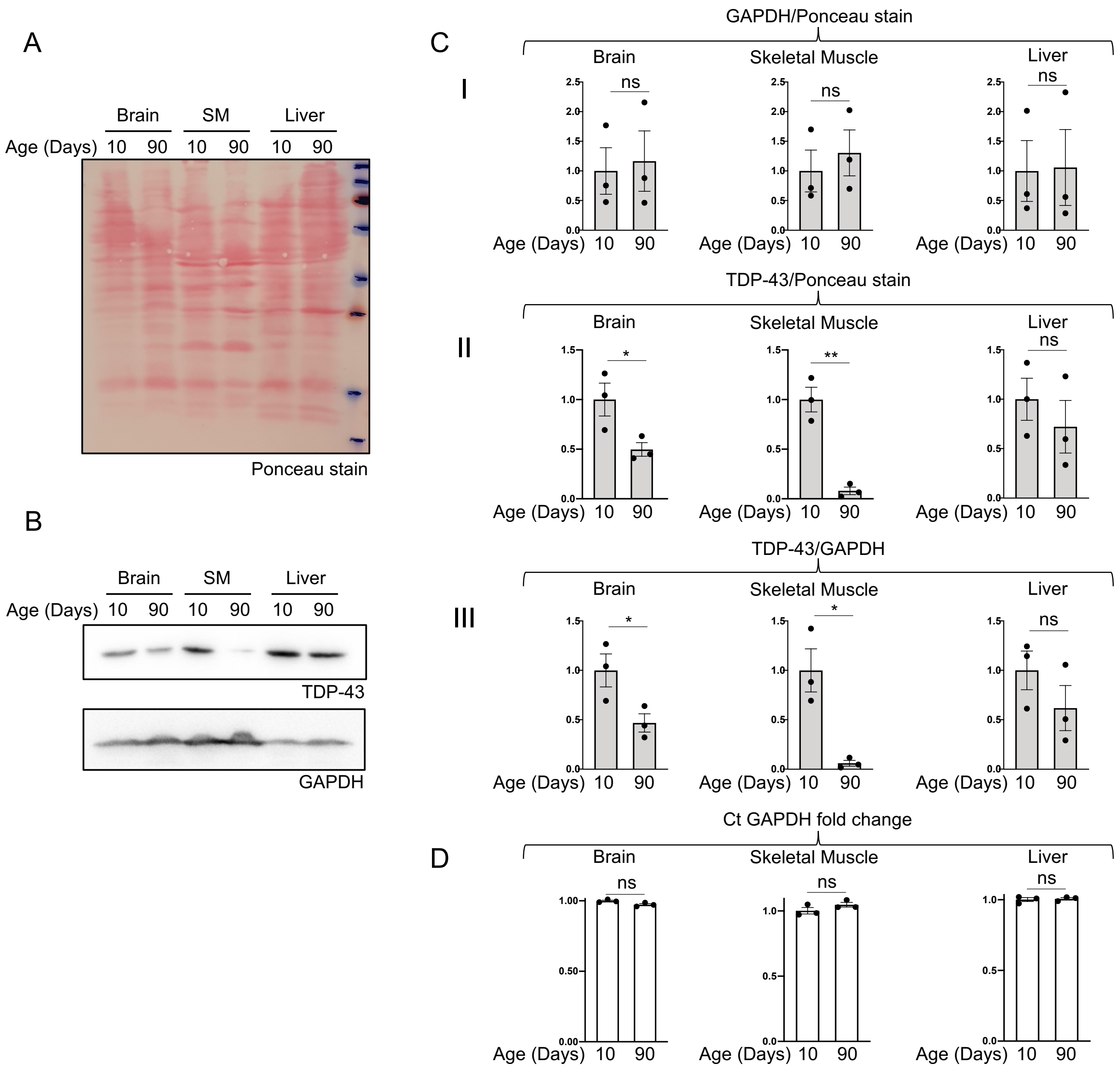

Fig. S1. (A) Representative ponceau stain used for quantifying total protein.(B) Representative western blot against TDP-43 and GAPDH. (CI) Relative expression of GAPDH in 10 and 90 days old tissues, normalized with total protein stain. (CII) Relative expression of TDP-43 in 10 and 90 days old tissues normalized with total protein stain.(CIII) Relative expression of TDP-43 in 10 and 90 days old tissues normalized with GAPDH.(D) Real time GAPDH Ct values in 10 and 90 days old tissues expressed as fold change over the 10 days value. Values are mean ± s.e.m. from 3 animals. Black circles represent individual data points. Pairwise comparison was performed with the two-tailed Student's t-test. $P \geq 0.05$ not significant (ns), $P < 0.05$ *, $P < 0.01$ **.

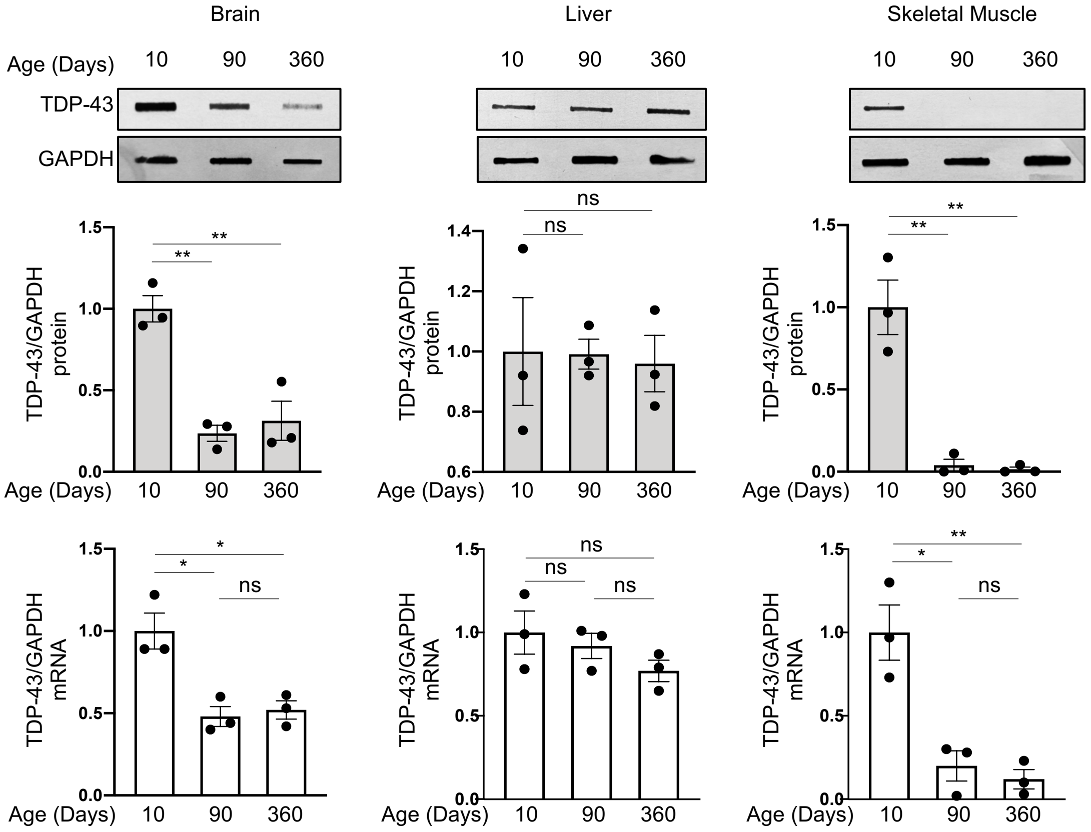

Fig. S2. Representative western blots showing the expression levels of TDP-43 between 10, 90 and 360 days in different mice tissues. Middle panel bar charts showing the relative protein levels, lower panel bar charts showing the relative mRNA levels. Values are normalized for GAPDH and expressed as fold over TDP-43 level in 10 days old tissues (n=3, two males and one female for each group. For qPCR data, two technical replicates for each of three biological replicates were performed). All values are mean ± s.e.m. Black circles represent individual data points. Pairwise comparison was performed with the two-tailed Student's t-test. P ≥ 0.05 not significant (ns), P< 0.05 *, P< 0.01 **.

Disease Models & Mechanisms • Supplementary information

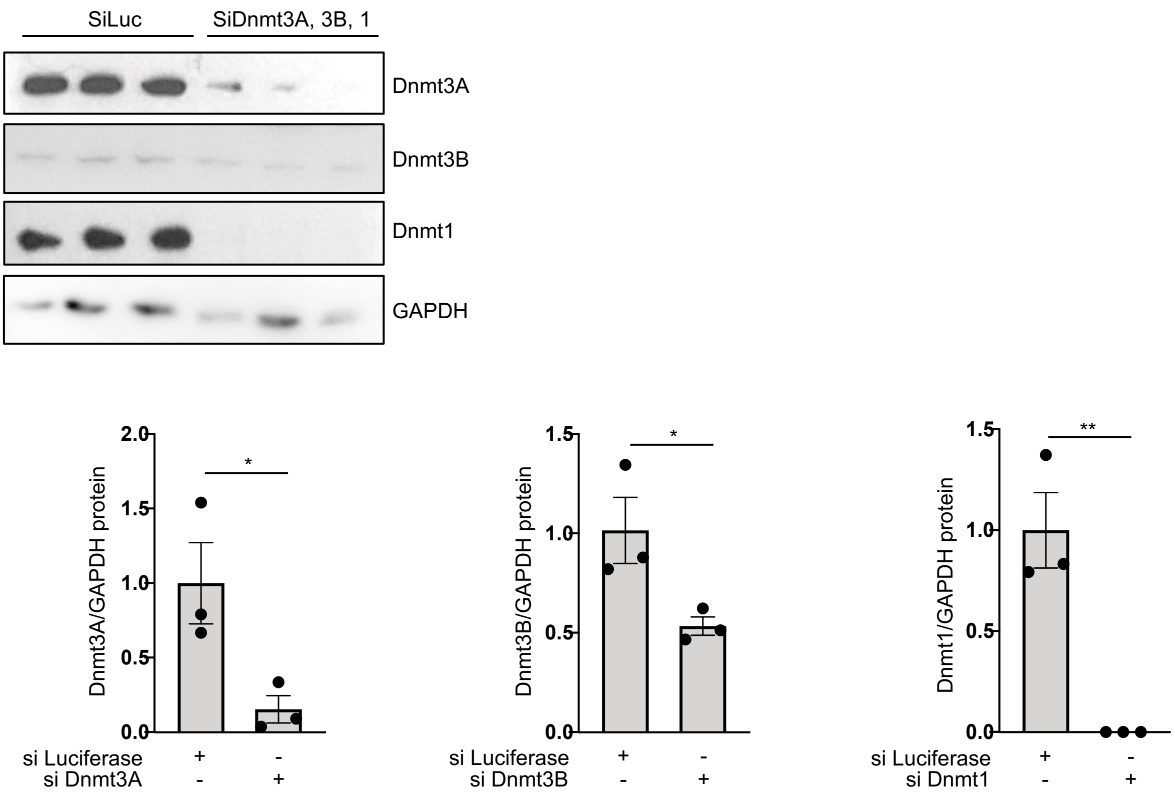

Fig. S3. Quantification of Dnmt3A, 3B and 1 silencing. Top panel shows western blots of Dnmt3A, 3B, 1 levels before and after siRNA treatment in NSC-34. Below, bar charts showing the quantification of each Dnmt after knockdown in respect to wildtype levels. Values are normalized for GAPDH and expressed as fold over wildtype levels. All values are mean ± s.e.m. (n=3). Black circles represent individual data points Pairwise comparison was performed with the two-tailed Student's t-test. $P \geq 0.05$ not significant (ns), $P< 0.05$ *.

Disease Models & Mechanisms • Supplementary information

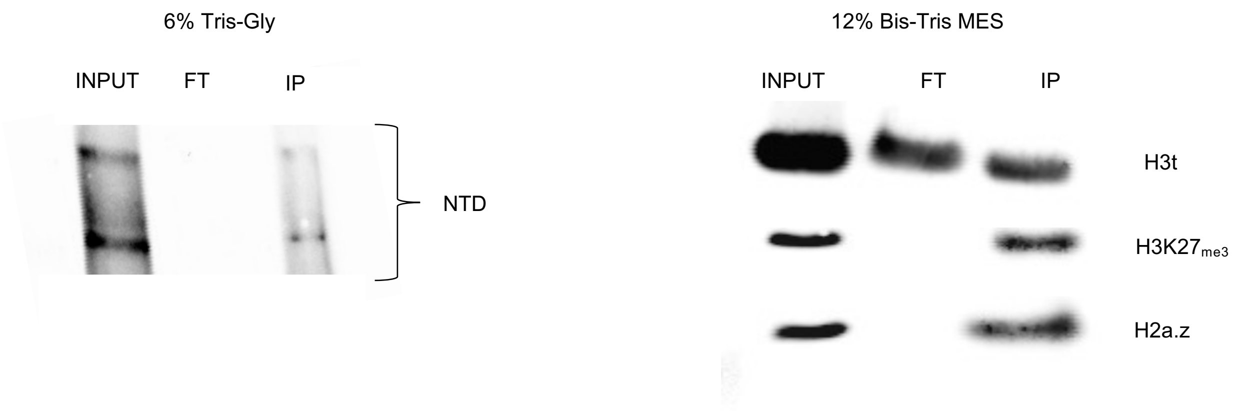

Fig. S4. ChIP antibody validation. ChIP with each antibody indicated on the right of the western blots was performed followed by a western blot with the same antibody. FT, flow through. IP, indicates the immunoprecipitation.

Disease Models & Mechanisms • Supplementary information

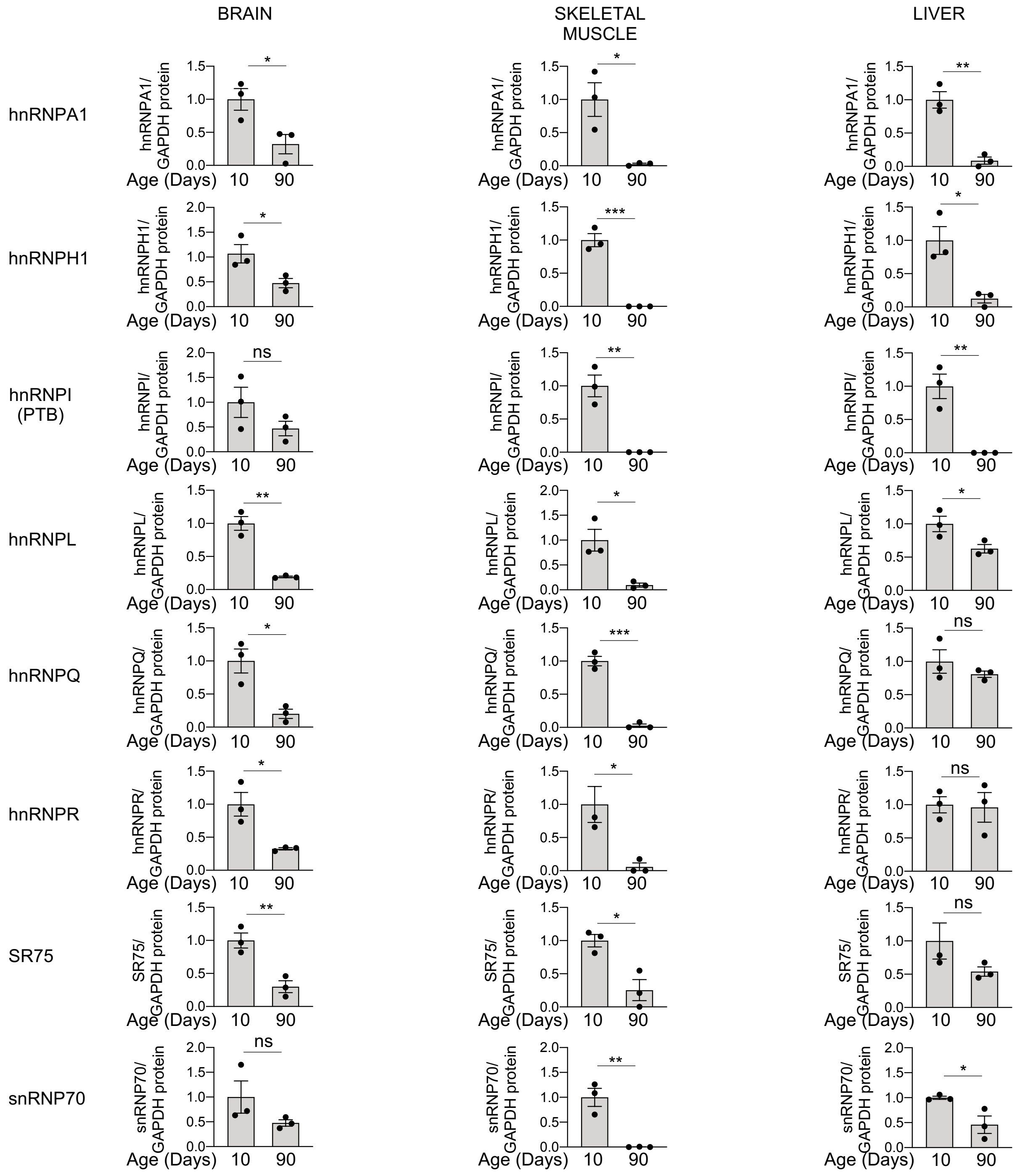

Fig. S5. Relative expression levels at 10 and 90 days in different mice tissues of proteins involved in RNA metabolism, names on left. Bar charts showing the relative expression. Values are normalized for GAPDH and expressed as fold over the protein level in 10 days old tissues (n=3;). All values are mean ± s.e.m. Pairwise comparison was performed with the two-tailed Student's t-test. P ≥ 0.05 not significant (ns), P< 0.05 *, P< 0.01 **. Black circles represent individual data points

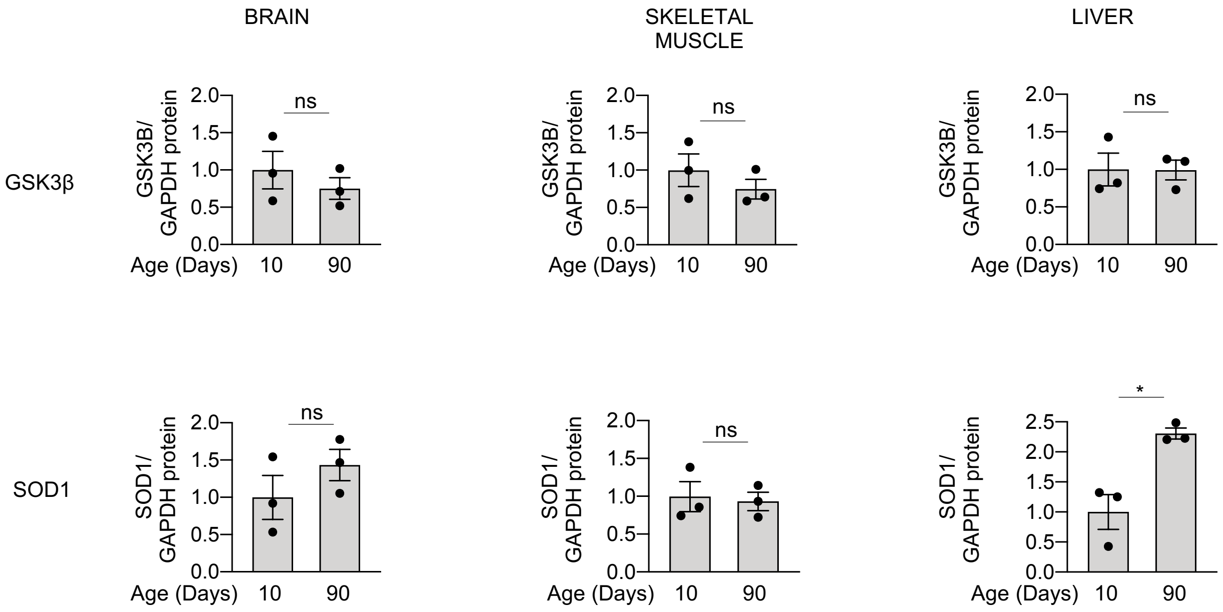

Fig. S6. Relative expression levels at 10 and 90 days in different mice tissues of proteins involved in cellular pathways other than RNA metabolism, names on the left. Bar charts showing the relative expression. Values are normalized for GAPDH and expressed as fold over the protein level in 10 days old tissues (n=3; two males and one female for each group). Black circles represent individual data points All values are mean ± s.e.m. Pairwise comparison was performed with the two-tailed Student's t-test. P ≥ 0.05 not significant (ns), P< 0.05 *.

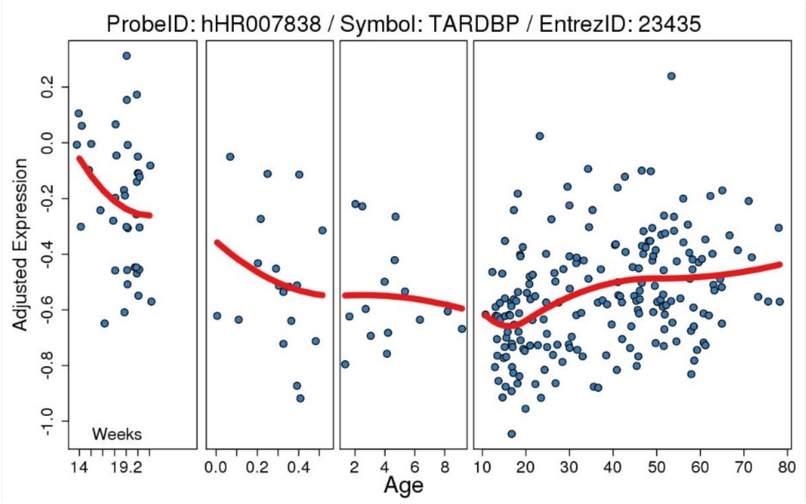

Fig. S7. Human TDP-43 expression data extracted from braincloud. Human brain RNA data (elaborated from Colantuoni et al., 2011), showing expression levels (y axis) with respect to age (x axis).

Disease Models & Mechanisms • Supplementary information
